# Indents

**Published:** 1913-04-15

**Authors:** 


### INDENTS.

**☞Brief Articles of Technical or Scientific Value will receive notice and comment. Contributions invited.**

*Instructions for Brushing the Teeth.* Teach the child the proper way to handle a brush. The essayist's plan is always to begin brushing on grinding surfaces of the back teeth, with a backward and forward motion, keeping the bristles pointed rootwise, rotating the brush from side to side, so that the bristles just miss the gums on both lingual and buccal surfaces; this is to clean the fissures. Then placing the brush well up on the gums, with a rolling motion, brush towards the occlusa surface, *i. e.*, up on the lower and down on the upper teeth, on both the lingual and buccal surfaces. When brushing the lingual surfaces of lower molars, the tongue should be drawn well back so as to expose those surfaces of the teeth to the brush. Frequently I give a demonstration showing the patient how to use a brush, and then have him practice before me until he uses it properly.

It is a very common thing for a patient to start brushing always in one place, usually at the gingival margins of some of the anterior teeth. This should be watched for and stopped, for while few do too much brushing, the brush is always stiffer when first put into the mouth, and this in connection with the first grit, if powder is used, applied in one place year after year, is to the gums and very thinly protected necks of the teeth what the constant dropping of water is to a stone.—F. H. Skinner in *Items of Interest.*

*The Gold Band in Crown Work.* I am led to believe that there is something very selfish back of the abuse which the gold band receives from many dentists. The greatest abuse which it does receive is the manner in which it is constructed. I have been told by some operators that they have not used a full cope in six, eight or ten years, in bridge work or porcelain crown work. When I hear that I always say to myself, there is another man piling up work for the other fellow.

The proper protection of small weak roots, and roots which are subjected to the particular strain and leverage which for instance comes upon the first bicuspids, makes the construction of a protective cope mechanically correct. I see continually enough of the disaster that follows in the wake of the bandless crown which has been placed where it is not indicated, and from the hands of men whom I would be inclined to credit with average or more than average care and skill, to believe that I have good ground for my position. Robert J. Cruise, in *Dental Review.*

*The Present Status of Free Dental Infirmary Work in Chicago.*

In addition to the Englewood free dental infirmary, which was one of the first started in Chicago, and which is still running and doing excellent service under the direction of the United Charities of Chicago and the Englewood Dental Society, there are at present ten infirmaries located in the public school buildings in different parts of the city where the children of the poor can receive dental service free of charge. These infirmaries are conducted by the Public Service Commission of the Chicago Dental Societies in conjunction with the Department of Health. The equipment was furnished by private contributions, mostly through the generosity of Mr. Julius Rosenwald, who is also paying the salaries of the operators. These infirmaries are open during school hours every school day in the year. The supplies are furnished by private contributions, and the rooms are heated and cleaned by the Board of Education. Inspection work is carried on by volunteer service from members of the dental societies under direction of the Department of Health, and already about 40,000 children have been examined, with a percentage of 95 defective.

The value of this infirmary service is being demonstrated daily. Principals of schools are loud in their praise of the movement, and acknowledge that it is already increasing the efficiency of the pupils. The waiting list at each infirmary is far in advance of the operator's capacity, and as the movement becomes more widely known the demand will be greater. During the month of February, 1913, more than five thousand operations were performed at these ten infirmaries, and already the amount of suffering that has been relieved has more than repaid for the money and energy invested.

As soon as sufficient funds can be raised a campaign of education will be started to enlighten the parents and children on matters of oral hygiene—this being really the most significant thing that can be done for the public welfare. To induce people to prevent disease is far preferable to curing the disease after it has occurred, and it is along these lines that the commission hopes to move, now that provision is being made for the relief of existing suffering.—Editorial in *Dental Review.*

*Filling Children's Teeth.* I understand the title of the paper was "Difficult Problems in Dentistry Solved by the Casting Method." Certainly it solves the problem of filling children's teeth. I have been singing this anthem to the profession for some time—the filling of children's teeth with gold. One said it was a difficult proposition. Another said it was too expensive; that parents would not pay the price of gold to fill their children's teeth. Both of those arguments, which were given to me all over this country, and in Canada, have been most beautifully exploded since then, because orthodontists now find it a great deal easier to get $1,500 out of a parent for regulating his child's teeth, than the family dentist does to get $15 for a whole set of teeth for the father. That is a fact that has not been appreciated. The orthodontist can tell you something about that, and I know, because I practice some orthodontia myself in addition to my other work.

I get just as much for an inlay in a child's tooth as for an adult's, and without complaint. It is a curious thing that I should be told to-night that no permanent fillings should be put into children's teeth, but that we should patch those teeth and wait until the children are responsible themselves. Not one of my children have come to me thus far this fall who has needed to have a tooth pulled, and it is because those particular children have all had inlays inserted in the morsal surfaces of their permanent molars, and because the inlays are doing permanent work.

I have never advocated that a tooth should be filled regardless of conditions, but where a temporary filling must be used, what I have said is "fill the teeth so temporarily that you will be obliged to fill them again, and let the patient understand that they are not filled permanently, but that they will have to come back to have them filled over again." There are only two things to put in a child's permanent molar—gutta percha, because it will not last as long as cement sometimes does, and gold; and I am putting in more gold now, because I can do it with less regard to strain. Many cases where I was obliged to use gutta percha year after year, or six months after six months, or three months after three months, I can fill now with the gold inlay at the first treatment; and it is not only the casting process that makes this possible, but the totally different shapes of the cavity. Cavities for inlays may be prepared with stones, and so may be prepared wet, and it makes it less painful. If any problem has been made easier, if any problem has been solved by Wm. H. Taggart, it is the difficulty of filling children's permanent teeth with permanent gold inlays, and I stand on this gold proposition with both feet now, instead of on one foot, as formerly.—Dr. Ottolengui in *Items of Interest.*

*A Possible Danger in Amalgam Fillings.* From a very extended clinical experience I have reached the conclusion that the use of a variety of amalgams in the same mouth produces the symptoms of distress that are totally absent if only one kind is used. On account of the variety of metals found in the various amalgam formulas these alloys become *incompatible*, and they show their incompatibility when bathed in the fluids of the mouth by setting up galvanic action. Mercury has too often received the blame which should really rest upon the shoulders of induced galvanism.

Mercury is necessarily the one most important ingredient, because there could be no amalgam without it, and when mixed with the alloy properly the mercury practically becomes inert. The subject of oral galvanism is either not understood or too much ignored by the members of the profession. If a lump of zinc and a lump of copper are placed separated from each other, in a saline fluid a decided electrical action ensues, yet daily dentists do not hesitate to similate such a condition in the mouth. One dentist places a copper amalgam filling and the next dentist one of silver, tin and zinc, and possibly the next dentist consulted uses still another mixture. Can you wonder at irritable nerves, diseased pulps, and sensitive peridental membranes? It is not the result of mercury, but the application of incompatible alloys by the unthinking dentist. Almost any two metals of the lower class, or one of the regal class and another of the lower class, when placed in the mouth, will under certain conditions produce galvanism. The condition is present even when the sensibility of the patient does not express it. If a mixture of the two metals or one metal and an alloy is made by placing them in the same filling there is the minimum of galvanic action. If the two metals are placed in different fillings and approximate or are in contact we have the maximum condition of galvanism. This applies in approximal fillings in the same jaw or in occluding surface fillings of opposite jaws. These well-understood clinical facts lead to the conclusion that the multiplicity of amalgams used in the same mouth is responsible for conditions that condemn the use of this variety of filling material, and that mercury, the much abused requisite of every amalgam, becomes an unobjectionable feature. Patients are frequently heard to remark that they have never had tooth pain until after they had received the services of a dentist. I believe that the placing of galvanic batteries in the mouth by inserting incompatible fillings is largely responsible for such a condition. I believe that the adoption of a *specific* amalgam to be used by all dentists is the logical antidote.

Many dentists have probably not had the experience detailed in this paper, but if they will investigate the matter the conclusion will be reached that it is because they have had the same patients for years, and have always adhered to the same alloy. Even if defective alloy is used I believe that greater salvage and more comfort is secured than if higher grade alloys of different composition are used in the same mouth. If a single high-grade alloy is used the maximum of salvage and comfort is secured. The public is daily becoming more intelligent about dental services and will be very quick to recognize incompatibilities in the mouth if the dentist will point them out. Superior discernment of cause of oral evils will quickly distinguish between the ethical practitioner and the indifferent charlatan. Prescriptions containing incompatible drugs quickly stamp the ignorant physician, so why not the dentist? Dental patients that have had a group of incompatible alloys cut out of their teeth and have received relief from painful symptoms by having the restoration made with compatible ones will gratefully recognize the superior services.

Patients are compelled to change dentists on account of removals from one city to another, or by death, but in such cases correspondence will reveal the alloy used where the patient has been a consistent one theretofore. If we cannot immediately establish a single specific alloy, such a procedure will at least be a move in that direction and elevate the profession to a higher plane.—Dr. Junkerman in March *Brief*.

*Bromural in Dentistry.* Bromural is a combination of isovalerianic acid with the isopropyl group and bromine. Bromural differs from the other valerian preparations in that it is narcotic. It possesses a mild odor and is altogether free from disagreeable by or after-effects. In dentistry, there is a great demand for a drug which in small dose will quiet the nerves, remove the fear and signs of restlesness, and check the secretions, and in larger doses will diminish the pains so intensely felt in weakened patients with inflammatory processes about the teeth and jaws and thus leads to a natural, refreshing sleep. Bromural meets all these requirements best, and can be given in doses of 5 to 10 grains where there is fear of operation, in pain after the introduction of arsenic and in neuralgic pains and pains owing to periodontitis, periostitis, lymphademitis. If given in doses of 10 grains before narcosis, the stage of excitation is dimished. In the severer grades of insomnia, a second dose of 5 grains may be given with good results after three hours.—*Zahnaerztl. Rundschau,* 1911, No. 50.

*Bridge Repair, Ouick, Without Removal.* A patient reported who had broken the central incisor from his bridge. His business engagements were such as to make it quite impossible to remove the bridge for repairs. I decided to use a Steel tooth and backing. With acarborundum wheel I removed the pins of the broken tooth and enough of the gold to make room for the Steel backing. It was the work of but a few minutes to make a groove in the backing of the adjoining teeth and slide the Steel backing into the same. After shaping the new tooth, I proceeded to cement the tooth and backing into place. The whole operation took about fifteen or twenty minutes, and has proved satisfactory and permanent.—Dr. Wm. Lowenthal, South Orange, N. J., *Dental Digest,* November, 1912.

